# Ultrasound-assisted modified paramedian technique for spinal anesthesia in elderly

**DOI:** 10.1186/s12871-022-01751-0

**Published:** 2022-07-30

**Authors:** Wei Zeng, Yisa Shi, Qihui Zheng, Shengfang Du

**Affiliations:** Department of Anesthesiology, Second Clinical Hospital of Lanzhou University, Lanzhou, Gansu Province China

**Keywords:** Spinal anesthesia, Modified paramedian technique, Ultrasound-assisted location, Elderly, Urological surgery

## Abstract

**Background:**

At present, there are two techniques which are widely applied clinically; the midline and the paramedian. Both methods are difficult for clinicians when treating the elderly. The aim of this work is to explore the feasibility of an ultrasound-assisted modified paramedian technique for spinal anesthesia in the elderly. This would provide clinicians with a new and easy-to-operate technique.

**Methods:**

A total of 150 elderly patients who were scheduled for urology surgery under spinal anesthesia in our hospital were randomly divided into three groups (*n* = 50): (i) midline technique group (group M), (ii) paramedian technique group (group P), and (iii) modified paramedian technique group (group PM). All spinal anesthesia were performed by the same second-year resident.

**Results:**

Compared with groups M and P, group PM had significantly higher first-attempt success rate (*P* < 0.05, especially in patients aged 65-74 years), fewer attempts (*P* < 0.05), and higher patient satisfaction score (*P* < 0.05). Compared with group M, the time taken to perform spinal anesthesia and the number of needle redirections were significantly reduced in group PM (*P* < 0.05). There was no statistically significant difference between groups PM and P. There were also no statistically significant differences in the cases of inconsistency between ultrasound-assisted and landmark-guided location of intervertebral space, the time taken to ultrasound-assisted location, the onset time to pain block at T_10_, the incidence of hypotension, anesthesia effect and the incidence of headache, lower back pain, or nausea and vomiting, within 24 h after surgery.

**Conclusions:**

The modified paramedian technique in spinal anesthesia for elderly patients can significantly improve the first-attempt success rate, reduce both the number of attempts and procedure time, and minimize tissue damage during the operation. Compared with the traditional techniques, the modified paramedian technique combines the advantages of both the midline and the paramedian methods, and is easy to learn. It is worthy of further research and application.

**Trial registration:**

Prospectively registered at the China Clinical Trial Registry, registration number ChiCTR2100047635, date of registration: 21/06/2021.

## Background

Due to an increasingly ageing population, the number of elderly who need surgical treatment under spinal anesthesia is increasing. The mean age of the spinal anesthesia cohort for hip fracture repair demonstrated a similarly increasing trend over time whereas the mean age of the general anesthesia cohort did not [1]. Studies have shown that the risk of many urinary system diseases (such as benign prostatic hyperplasia, bladder tumors, or urethral stricture) increase drastically with ageing. Even for patients who have undergone urethroplasty, the decline of detrusor function related to age may cause repeat urinary difficulties [2–4]. Compared with general anesthesia, spinal anesthesia has been recognized as providing greater hemodynamic stability, higher patient satisfaction, lower rate of opioid use, higher rate of opioid-free recovery, and lower maximum Post-Anesthesia Care Unit (PACU) pain scores [5–7].

Due to the degeneration of the lumbar spine (bone hyperplasia, ligament calcification, intervertebral space stenosis, spinous process hyperplasia, etc.) caused by ageing, spinal anesthesia in elderly patients might be difficult [8–11], especially for inexperienced residents. There are two traditional techniques for spinal anesthesia (midline technique and paramedian technique), each of which has advantages and disadvantages in terms of operation and application. Ultrasound (US) can be safely applied to aid spinal anesthesia of elderly patients, but with varying efficacy. Park et al. [12] showed that pre-procedural ultrasound can improve the first success rate of spinal anesthesia in elderly patients from 17.5 to 65%. While Rizk et al. [13] found that when performed by junior residents a pre-procedural US scan did not improve the ease of midline or paramedian spinal anesthesia in the elderly when compared to the conventional landmark technique. Therefore, the aim is to combine the advantages of two traditional methods, improve the success rate of spinal anesthesia, reduce the time taken to puncture and the number of attempts, and to facilitate the process of anesthesia in elderly patients.

The purpose of our study is to explore the feasibility of ultrasound-assisted modified paramedian technique for spinal anesthesia in elderly when compared with midline and paramedian techniques. We hypothesized that an ultrasound-assisted modified paramedian technique for spinal anesthesia may concentrate the advantages of both. This study therefore shows the results of a prospective, randomized, controlled study in our hospital.

## Methods

This study was approved by the Ethics Committee of the Second Hospital of Lanzhou University (2021A-106) and was registered on 21/06/2021, at the China Clinical Trial Registration Center (http://www.chictr.org.cn/; registration number ChiCTR2100047635). Patients undergoing urological surgery under spinal anesthesia were consecutively enrolled at the hospital from April 2021 to September 2021. Each patient signed a written consent the day before surgery. Inclusion criteria were as follows: age ≥ 65 years old, and American Society of Anesthesiologists physical status I to III. Patients were excluded if they refused participation, had contraindications to spinal anesthesia (uncertain neurological disorders, local infections, allergic to local anesthesia, coagulopathy), had spinal deformities, had a history of spinal surgery, were problematic for ultrasound scanning, or having communication difficulties.

The patients were randomly allocated into three groups using a computer generated randomized number table. The groups assigned were (i) midline technique group (group M), (ii) paramedian technique group (group P), and (iii) modified paramedian technique group (group PM). The related information was placed in an opaque envelope, which was numbered 1-150 according to the order the patient was included, so that the envelopes corresponded to the patients one by one. Considering the feasibility of the study, only patients were blinded to their treatment group.

After the patient entered the operating room, invasive or non-invasive blood pressure, pulse oximetry, and three-lead electrocardiography were routinely monitored. Peripheral venous access was established. All patients were placed in a standard lateral recumbent position with an oxygen inhalation face mask at a flow rate of 2-3 L/min. The physician responsible for performing the anesthesia throughout the study was a resident. Initially the resident palpated the surface anatomical landmarks with the conditional identification (the intersection between the highest point of the iliac crest and the spine is regarded as the L3-4 spinous process space or the L4 spinous process). Next, the site of needle insertion was marked on the surface of the patient’s skin. Throughout the study, a senior anesthesiologist who was proficient in the use of ultrasound performed the ultrasound-assisted location. The Sonosite (M-TURBO, NASDAQ: SONO, USA) with a low frequency (2 to 5 MHz), curved array probe, and a depth of 9.2 cm was used. The probe was placed at parasagittal oblique (PSO) plane, 1–2 cm from the midline. On the screen the sacrum is seen as a continuous bright line of high echo. Moving the probe upwards from the sacrum, the structure of the articular process with a “hump sign” could be seen. Once the L_3-4_ intervertebral space was identified, the probe was tilted slightly until an optimal image of the anterior/posterior complex appeared (the anterior complex is the imaging of vertebral body and posterior longitudinal ligament, and the posterior complex is the imaging of *ligamentum flavum* and dural sac). Shifting the image to the center of the screen, a line perpendicular to the midpoint of the long axis of the probe was drawn on the surface of the patient’s skin with the guidance of the “M-Mode”. This line was regarded as the L_3-4_ intervertebral space. Next, the probe was rotated 90° at the transverse midline (TM) plane, scanning from cephalic to caudal in the space to determine adjacent spinous processes. The spinous processes, articular processes, and anterior and posterior complexes, were visible by slightly adjusting the probe. The two points corresponding to the spinous processes were marked on the surface of the skin. The line connecting the points was regarded as the posterior midline of the spine. Finally, the intersection of the two connecting lines was identified as the needle insertion point “O” of group M. In group P, the injection site was 1.5 cm lateral to the “O” on the right of the midline, and the needle punctured the skin at an angle of about 75°. In group PM, the site was 0.5 cm lateral to the “O” on the right of the midline. For both groups M and PM, the needle was perpendicular to skin.

Once the site had been identified all the lines were erased. The resident was supervised to perform a strictly aseptic technique throughout the process. A 22-guage ‘Quincke’ spinal needle was used for the puncture. After making sure the cerebrospinal fluid flowed out smoothly, 2 ml of 0.75% ropivacaine was injected into the subarachnoid space with the slope of needle cephalad at a speed of about 0.2 ml/s. After the injection was completed, the patient was immediately placed in a supine position. The level of sensory block was adjusted up to T_10-8_. During surgery, the patient’s blood pressure was maintained within a range of ±20% of the baseline. Failure was defined as: > 3 attempts times, > 10 needle redirections, the replacement of the puncturing gap, or the change of anesthesic method, and would result in cessation of the trial.

### Data collection

The primary outcome was the first attempt success rate. Secondary outcomes were the number of attempts (each time when the needle was returned to the subcutaneous to readjust the direction of the needle to puncture again or the needle leaved the skin to puncture again was considered another separate attempt), the number of needle redirections, the cases of inconsistency between ultrasound-assisted and landmark-guided location of intervertebral space, the time taken to perform spinal anesthesia (the time from the needle puncturing into the skin after local anesthesia to free flow of cerebrospinal fluid), patient satisfaction (1-5 points), the time taken to ultrasound-assisted location, the onset time to pain block at T_10_, the effect of anesthesia, the incidence of headache, back pain, and nausea and vomiting within 24 h after the operation. All data was collected by an anesthesiologist who did not take part in the administration of patients.

### Statistical analysis

The sample size was counted by PASS15 software (NCSS, America). In the study hospital, the first attempt success rate in the elderly using the midline technique was 53.6%, and with the modified paramedian technique 79.3%. To determine the sample size we looked at a previous study [10], in which the first attempt success rate in the elderly with the paramedian technique performed by residents was 42.0%. With an α error of 5%, a β error of 10% (90% power) and dropouts of 10%, a sample size of 50 patients per group was required.

Statistical analysis was performed using SPSS Version 25 (IBM, Armonk, NY). The measurement data were tested for normality. Normally distributed data were presented as mean ± standard deviation (x ± s), and the comparison between groups was performed using an ANOVA test. The non-normally distributed data were described as the Median (M) and Interquartile range (IQR) and were compared between groups using Kruskal and Wallis tests. Counting data were presented as numbers and percentage (n, %) and analyzed by Chi square or Fisher’s exact test. *P* < 0.05 was considered statistically significant.

## Results

**Fig. 1:**
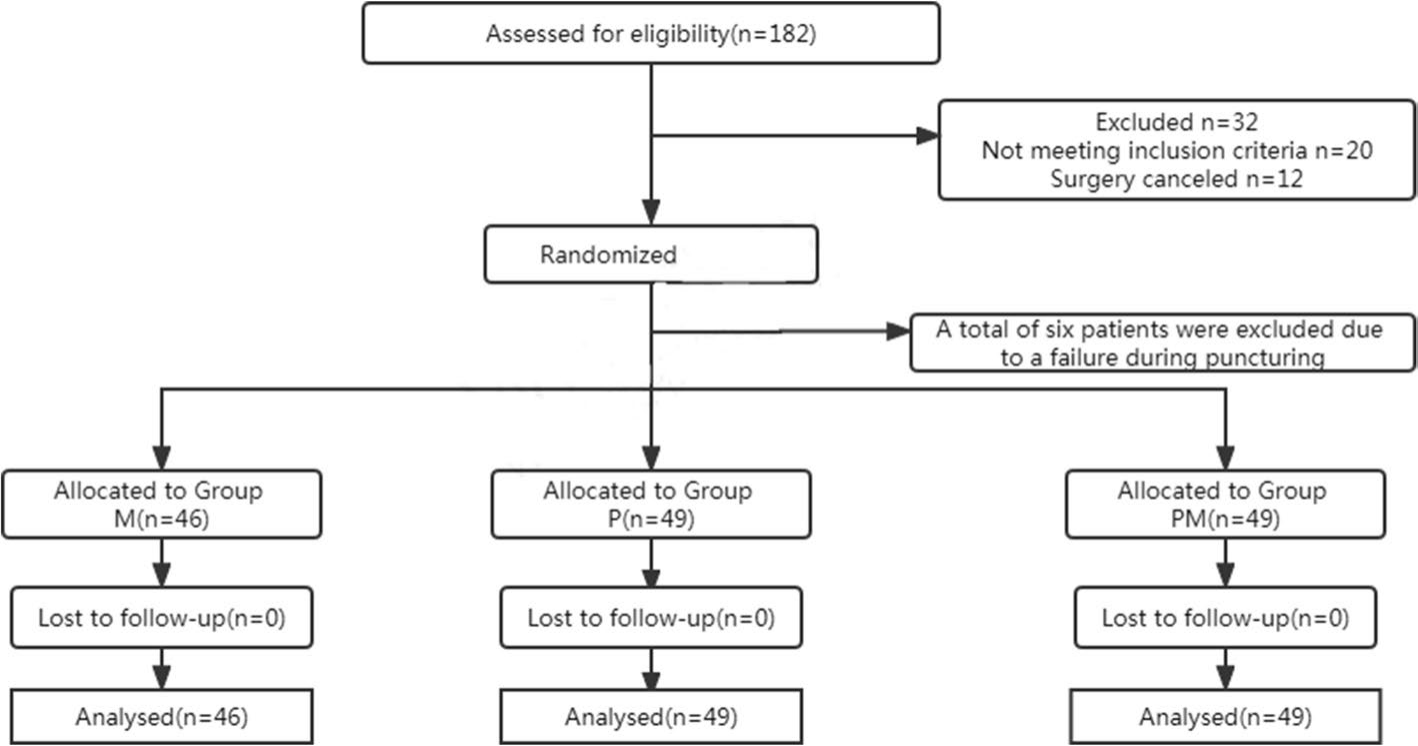
Consort flow diagram

A total of 150 patients were initially enrolled in the study, of which six cases (four in group M, one each in groups P and PM) were excluded due to failure during puncturing. No treatment was converted to general anesthesia. There were no dropouts in the study, and the remaining 144 cases were included in the final analysis (Fig. 1). There were no statistically significant differences in age, height, weight, BMI, time of surgery, gender, American Society of Anesthesiologists classification, or type of surgery among the three groups of patients (Table 1).

**Table 1:** Comparisons of characteristics of the three groups

	Group M (*n* = 46)	Group P (*n* = 49)	Group PM (*n* = 49)	*P*
Age (y)	72.33 ± 7.02	71.49 ± 6.53	72.10 ± 5.53	0.802
Height (cm)	168.78 ± 5.49	169.94 ± 6.64	168.35 ± 5.25	0.380
Weight (kg)	64.15 ± 9.49	65.24 ± 10.30	66.51 ± 10.89	0.534
BMI (kg·m^−2^)	22.51 ± 3.08	22.55 ± 3.03	23.42 ± 3.28	0.276
Time of surgery (min)	97.15 ± 47.25	82.12 ± 36.81	98.33 ± 42.93	0.114
Gender				0.371
M	46(100)	47(95.9)	46(93.9)	
F	0(0)	2(4.1)	3(6.1)	
ASA				0.805
I	3 (6.5)	2 (4.1)	3 (6.1)	
II	36 (78.3)	42 (85.7)	42 (85.7)	
III	7 (15.2)	5 (10.2)	4 (8.2)	
Type of surgery				0.741
TURP	21 (45.7)	21 (42.9)	28 (57.1)	
TULIP	7 (15.2)	8 (16.3)	8 (16.3)	
Ultrasound-guided prostate biopsy	7 (15.2)	10 (20.4)	5 (10.2)	
Others	11 (23.9)	10 (20.4)	8 (16.3)	

Values are given as mean ± SD or numbers (%)

*Abbreviations*: *TURP* Transurethral Resection of Prostate, *TULIP* Transurethral Ultrasound-guided Laser Induced Prostatectomy, *SD* standard deviation

There were no statistically significant differences in the data related to ultrasound-assisted location (Table 2).

**Table 2:** Comparisons of data related to ultrasound scanning of the three groups

	Group M (*n* = 46)	Group P(*n* = 49)	Group PM (*n* = 49)	*P*
Cases of inconsistency between ultrasound-assisted and landmark-guided location of intervertebral space	14 (30.4)	12 (24.5)	8 (16.3)	0.266
Time taken to ultrasound-assisted location	139.57 ± 31.18	144.22 ± 36.54	144.43 ± 33.50	0.734

Values are given as mean ± SD or numbers (%)

The first-attempt success rate of the three groups was 47.8% (Group M), 42.9%, (Group P) and 77.6% (Group PM). This was significantly higher in group PM than groups M (*P* < 0.05) or P (*P* < 0.001). The number of attempts in group PM (1[1-1]) was significantly fewer than groups M (2[1-2]) or P (2[1-2]), *P* < 0.05. The time taken to perform spinal anesthesia in group PM was significantly shorter than group M (*P* < 0.05), but was not different from group P. The number of needle redirections in group PM (1[0-2]) was significantly fewer than group M (2[1-3.25], *P* < 0.05), but there was no statistically significant difference with group P (1[1-2]). The patient satisfaction score during the process of puncturing was higher in group PM (4[4-5]) than groups M (4[3-4], *P* < 0.05) or P (4[3.5 -4], *P* < 0.05). Compared with group M, the time taken to perform spinal anesthesia in group P was significantly reduced (*P* < 0.05), and the other related data during puncturing showed no statistical significance. There was no statistically significant difference in the onset time to pain block at T_10_ among the three groups (Table 3).

**Table 3:** Comparison of data during anesthesia process of the three groups

	Group M(*n* = 46)	Group P (*n* = 49)	Group PM (*n* = 49)	*P*
First-attempt success rate	22(47.8)	21(42.9)	38(77.6)^ab^	0.001
Number of attempts	2[1-2]	2[1-2]	1[1-1]^ab^	0.003
Number of needle redirections	2[1-3.25]	1[1-2]	1[0-2]^a^	0.003
Time taken to perform spinal anesthesia	116.00[49.75-244.00]	68.00[44.50-129.50]^c^	56.00[36.50-106.00]^a^	0.001
Satisfaction scores	4[3-4]	4[3.5-4]	4[4-5]^ab^	0.001
The onset time to pain block at T_10_(s)	146.33 ± 26.63	145.08 ± 23.08	146.67 ± 24.35	0.945

Values are given as mean ± SD, median (IQR), or numbers (%)

^a^Group PM vs Group M

^b^Group PM vs Group P

^c^Group P vs Group M

To explore the influence of age on puncturing related outcome, subgroup analysis was conducted. All patients were divided into two groups: 65-74 years old and ≥ 75 years old. The results showed that there was no statistical significance in the composition ratio of the patients in the three groups (*P* > 0.05). For patients aged 65-74 years, the first-attempt success rate in group PM was significantly higher than groups M and P (*P* < 0.05). Compared with group M, the number of needle redirections in group PM was significantly reduced (*P* < 0.05), and the difference was not statistically significant when compared with group P. The number of attempts in group PM was significantly fewer than group P (*P* < 0.05), although the difference was not statistically significant compared with group M. There was no significant difference in satisfaction score among the three groups (*P* > 0.05). For patients ≥75 years old, there were no significant differences in the first-attempt success rate, the number of attempts, or the number of needle redirections among the three groups. However, the patient satisfaction score in group PM was significantly higher than groups M and P. For patients in both age groups, the time taken to perform spinal anesthesia in group PM was significantly shorter than groups M (*P* < 0.05), but there was no statistically significant difference when compared with group P (Table 4).

**Table 4:** Comparison of related data during puncturing in different ages among the three groups

	Group M	Group P	Group PM	*P*
Number of patients in different age groups				0.970
65-74 years old	31 (67.4)	33 (67.3)	34 (69.4)	
≥ 75 years old	15 (32.6)	16 (32.7)	15 (30.6)	
First-attempt success rate				
65-74 years old	17 (54.8)	15 (45.5)	27 (79.4)^ab^	0.014
≥ 75 years old	5 (33.3)	6 (37.5)	11 (73.3)	0.053
Number of needle redirections				
65-74 years old	2[0-3]	1[1-2]	0[0-1]^a^	0.015
≥ 75 years old	3[1-5]	2[0.25-3.75]	2[0-2]	0.110
Number of attempts				
65-74 years old	1[1-2]	2[1-2]	1[1-1]^b^	0.032
≥ 75 years old	2[1-3]	2[1-2]	1[1-2]	0.060
Time taken to perform spinal anesthesia(s)				
65-74 years old	105.00[43.00-147.00]	67.00[42.50-120.50]	54.00[29.75-63.50]^a^	0.014
≥ 75 years old	162.00[73.00-311.00]	70.00[47.50-160.00]	103.00[39.00-127.00]^a^	0.040
Satisfaction scores				
65-74 years old	4[3-4]	4[4-4]	4[4-5]	0.115
≥ 75 years old	3[3-4]	4[3-4]	4[4-5]^ab^	0.001

Values are given as median (IQR), or numbers (%)

^a^Group PM vs Group M

^b^Group PM vs Group P

There were no statistically significant differences in the effect of anesthesia and the incidence of nausea or vomiting, headache, or lower back pain within 24 h after surgery among the three groups (Table 5).

**Table 5:** Comparisons of the effects of anesthesia and postoperative adverse reactions within 24 h after surgery among the three groups

	Group M(*n* = 46)	Group P(*n* = 49)	Group PM (*n* = 49)	*P*
the effect of anesthesia				
I	44 (95.7)	48 (98.0)	48 (98.0)	0.386
II	2 (4.3)	1 (2.0)	0 (0)	
III	0 (0)	0 (0)	1 (2.0)	
Postoperative adverse reactions				
Nausea or vomiting	1 (2.2)	3 (6.1)	3 (6.1)	0.700
Headache	2 (4.3)	2 (4.1)	1 (2.0)	0.868
Low back pain	3 (6.5)	2 (4.1)	2 (4.1)	0.796

Values are given as numbers (%)

## Discussion

To effectively solve the difficulty of spinal anesthesia during the procedure in elderly patients a randomized controlled study examined the application of a pre-procedural ultrasound modified paramedian technique performed by residents, and compared it with two traditional techniques. The results showed that the modified paramedian technique proved more efficacious than the midline or paramedian techniques. The PM group had a significantly higher first-attempt success rate and fewer total attempts compared to the others.

Chen et al. [14] showed that compared with the paramedian approach, the modified paramedian approach had a higher first-attempt success rate, greater patient satisfaction, and fewer attempts in pregnant women. This was therefore a feasible approach for a resident, which was consistent with our study. The reasons may be as follows: (1) Compared to the paramedian technique, the injection site in the modified paramedian technique is closer to the midpoint of the spinous process space, and the path of needle insertion is shorter. For residents with less clinical experience, the angle and depth of needle insertion are easier to grasp, (2) Compared to the midline approach, it bypasses the bony three-dimensional structure of the lumbar spine to a certain extent, deviates from spinous processes, and weakens the influence of the intervertebral space stenosis on puncturing, (3) The needle is walked superiorly on the lamina and advanced into the interlaminar space through the ligamentum flavum [15], (4) Ultrasound scanning in the para-midsagittal position could obtain a larger intervertebral space and a clearer field of ligamentum flavum and epidural vessels [16]. Overall, the modified paramedian technique combines the advantages of two traditional techniques, making it more feasible to apply in spinal anesthesia for the elderly.

For nearly 10 years of clinical practice, we have found that even in cases of repeated failure for senior anesthesiologists to perform spinal anesthesia in the elderly the application of modified paramedian technique often gets success. Furthermore, compared with the midline technique, operators often feel a significant reduction in resistance in the process of needle insertion, especially in elderly patients. The reason for this may lie in the fact that the modified paramedian technique bypasses calcified supra-spinal and inter-spinous ligaments or the dense and tough part of ligaments of elderly patients which reduces the difficulty of puncturing. At the same time, it is especially suitable for residents with less experience since the insertion angle and the depth of the needle are easy to master. Previous studies [17–19] have shown that in the modified paramedian technique the needle tip can theoretically reach the subarachnoid space smoothly by setting the injection site 0.5 cm lateral to the midpoint of the L3-4 spinous process space with the needle perpendicular to skin.

Currently the midline and paramedian techniques are widely applied for spinal anesthesia in the elderly. In contrast to previous studies [13, 20, 21], our work showed that in the paramedian technique group only the time taken to perform spinal anesthesia was significantly reduced. The reason could lie in the different definitions of first-attempt success rate, and the practical abilities of the operators.

In order to explore the effects of the three techniques in patients of different ages, all patients were divided into two groups according to age for subgroup analysis. It was found that there were some differences between patients aged 65-74 years and those ≥75 years old. This may be related to the uneven distribution of the two age groups. The latter only accounted for approximately 30% of the total number of patients. The differences in patient life experience, education level, psychological state, and other aspects, may account for a higher satisfaction score in patients over 75 years old. In addition, through intra-group comparison, when using the same technique, the first-attempt success rate decreased with increasing age, while the number of needle redirections, number of attempts, and puncturing time increased. This indicated that there was a certain correlation between age and the difficulty of lumbar puncture in elderly patients, which is consistent with previous research [22].

Our study did, however, have some limitations. Firstly, the injection site was 0.5 cm lateral on the right of the midline in the test. Previous studies reported that the thickness and density of ligaments on both sides of the midline were different. *Ligamentum flavum* was significantly thicker on the right side [23, 24]. We did not explore whether it was better to identify the site as 0.5 cm lateral on the left of the midline or not, which is worthy of a further study. Secondly, the study did not apply real-time ultrasound guidance technique, which could be superior to an ultrasound-assisted technique due to full visualization. Thirdly, we felt the reduced resistance during the procedure of insertion, but we didn’t explore structures that the needle passed through; there has been no consensus for the reason up to now. Lastly, we only collected data regarding post-operative adverse reactions within 24 h after surgery. This may have increased if longer term data had been collected.

## Conclusion

In summary, the modified paramedian approach has certain advantages in spinal anesthesia performed by residents on the elderly. This includes a higher first attempt success rate, greater patient satisfaction, fewer attempts, and a shorter procedure time. This could therefore provide a reference for clinical practice. However, there are still very few related clinical studies on the approach, and further studies are still needed.

## Data Availability

The datasets used and/or analyzed during the current study are available from the corresponding author on reasonable request.
